# MFSleepNet: An Interactive Multimodal Fusion Framework for Automatic Sleep Staging

**DOI:** 10.3390/s26103085

**Published:** 2026-05-13

**Authors:** Ranran Gui, Chen Wang, Qunfeng Niu, Li Wang

**Affiliations:** 1 School of Electronic and Electrical Engineering, Zhengzhou University of Science and Technology, Zhengzhou 450064, China; 2125021@zit.edu.cn (R.G.); 2125020@zit.edu.cn (C.W.); 2 College of Electrical Engineering, Henan University of Technology, Zhengzhou 450001, China

**Keywords:** sleep staging, physiological signal processing, EEG and EOG fusion, multimodal learning, subject-specific adaptation

## Abstract

**Highlights:**

**What are the main findings?**

- MFSleepNet consistently outperforms representative single-modality and multimodal sleep staging methods on the Sleep-EDF, SHHS, and HSP datasets.
- The interaction-based EEG–EOG fusion mechanism improves multimodal feature representation and sleep stage classification performance.
- The gated temporal-channel attention module enhances discriminative temporal and channel-wise physiological patterns.
- Cross-subject evaluation reveals substantial inter-subject variability and limited baseline generalization on unseen subjects.

**What are the implications of the main findings?**

- Structured inter-modality interaction is beneficial for multimodal physiological signal fusion in sleep staging.
- Subject-specific adaptation can effectively improve personalization performance with limited labeled data.
- Inter-subject variability remains a major challenge for practical sleep staging systems and motivates future research on more robust subject-independent modeling.

**Abstract:**

Accurate automatic sleep staging remains challenging due to complex temporal dynamics, inter-subject variability, and the difficulty of effectively integrating heterogeneous physiological signals. Electroencephalogram (EEG) and electrooculogram (EOG) recordings provide complementary information for sleep analysis; however, most existing multimodal approaches rely on simple feature concatenation, which limits their ability to capture structured inter-modality relationships. This paper proposes MFSleepNet, a multimodal sleep staging framework that explicitly models interactions between EEG and EOG signals. The proposed system incorporates a multimodal feature fusion module to enable bidirectional information exchange between modality-specific representations, followed by a gated temporal-channel attention mechanism to adaptively emphasize informative temporal segments and signal channels, facilitating joint representation learning while preserving modality-specific characteristics. Experiments on three public datasets (Sleep-EDF, SHHS, and HSP) under an epoch-level cross-validation protocol show that MFSleepNet consistently outperforms representative single-modality and multimodal baseline methods in terms of overall accuracy, Cohen’s κ, and Macro-F1. Ablation studies further demonstrate the contribution of each functional module. Correlation analysis indicates stage-dependent variations in EEG–EOG relationships, while interaction-based experiments show that explicit feature interaction improves both joint and modality-specific representations. Grad-CAM visualizations provide interpretability of model decisions. External validation on unseen subjects reveals a noticeable performance drop, highlighting the challenges of inter-subject variability and the limited baseline generalization capability of the model. To address this, a lightweight subject-specific adaptation strategy is introduced, which improves performance using a small amount of labeled subject-specific data. Overall, the proposed framework provides an effective and interpretable solution for multimodal sleep staging while emphasizing the importance of structured inter-modality interaction and subject-adaptive modeling in practical applications.

## 1. Introduction

Accurate measurement of sleep stages plays a critical role in clinical sleep assessment and long-term physiological monitoring. Disruptions in sleep architecture are closely associated with metabolic dysfunction, cognitive impairment, and increased risks of cardiovascular and neuropsychiatric disorders [1,2]. Reliable and automated sleep staging is therefore essential for scalable sleep health evaluation in both clinical and home-based monitoring scenarios.

Polysomnography (PSG) remains the standard instrumentation for sleep assessment, providing synchronized recordings of multiple physiological signals throughout the sleep cycle [3]. According to the American Academy of Sleep Medicine (AASM), sleep is conventionally categorized into five stages: Wake (W), N1, N2, N3, and rapid eye movement (REM), each characterized by distinct neurophysiological patterns [4]. However, manual PSG-based scoring is labor-intensive and subject to inter-scorer variability, motivating the development of automated sleep staging systems with improved objectivity and reproducibility [5,6].

Recent advances in deep learning have enabled end-to-end modeling of physiological signals for automatic sleep staging [7]. Nevertheless, most existing approaches primarily rely on electroencephalogram (EEG) recordings, while complementary modalities such as electrooculogram (EOG) are often underutilized [8,9]. This single-modality paradigm limits measurement robustness, particularly for transitional stages with overlapping EEG characteristics, such as N1 and REM. In contrast, EOG signals capture eye-movement dynamics that provide discriminative information for specific sleep stages, indicating that EEG and EOG encode complementary and stage-dependent physiological characteristics [10].

Motivated by this observation, multimodal signal fusion strategies have been explored to improve sleep staging performance [11,12,13]. However, most existing multimodal frameworks employ shallow fusion schemes, in which modality-specific features are extracted independently and combined through simple concatenation. Such approaches inadequately capture structured inter-modality relationships arising from differences in temporal dynamics, phase alignment, and noise characteristics between EEG and EOG signals, thereby limiting measurement accuracy and generalization under subject-independent conditions [14,15].

To address these limitations, this study proposes a multimodal sleep staging framework, termed MFSleepNet, which jointly models EEG and EOG signals through an interaction-based fusion strategy and a gated temporal-channel attention mechanism. Unlike conventional multimodal approaches that rely on independent feature extraction followed by simple concatenation, the proposed framework introduces a cross-channel interaction mechanism that enables bidirectional modulation between EEG and EOG representations during the fusion process. In addition, a gated temporal-channel attention mechanism is adopted to refine the fused representation by adaptively weighting temporal and channel-wise information, thereby enhancing discriminative patterns associated with sleep dynamics. This design aims to provide a more effective and flexible solution for multimodal physiological signal integration.

The main contributions of this work are summarized as follows:

(1) A multimodal sleep staging framework is proposed that incorporates cross-modality interaction between EEG and EOG signals, moving beyond conventional fusion strategies based on independent processing and simple concatenation.
(2) An interaction-based multimodal fusion module is developed to model cross-modal dependencies via bidirectional channel-wise modulation between EEG and EOG representations.
(3) A gated temporal-channel attention block is introduced to refine the fused features by adaptively weighting temporal and channel-wise information, thereby enhancing discriminative patterns for sleep stage classification.
(4) Extensive validation on multiple public datasets under an epoch-level cross-validation protocol demonstrates improved classification performance.

## 2. Related Work

### 2.1. Single-Modality Sleep Staging Methods

Early automatic sleep staging research predominantly relied on single-modality EEG signals, and a substantial body of recent work continues to focus on single-channel EEG due to its practicality, ease of acquisition, and suitability for wearable applications. Representative studies have explored increasingly sophisticated architectures to enhance temporal modeling and feature representation. For instance, Huang et al. proposed a CNN-based model with SENet and HMM decoding, achieving an overall accuracy of approximately 84.6% on the Sleep-EDF dataset [16]. Eldele et al. introduced AttnSleep, which integrates multi-resolution convolutions with attention-based temporal encoding, reporting accuracies of around 81.3% on Sleep-EDF and 84.2% on SHHS [17]. Zhao et al. further incorporated RNN-based sequence modeling in SleepContextNet, achieving approximately 82.7% on Sleep-EDF and 86.4% on SHHS [18]. More advanced hybrid architectures, such as MultiScaleSleepNet and SleepHybridNet, further improve performance, reaching around 88.6% and 88.19% on Sleep-EDF, respectively [19,20]. Despite these improvements in overall accuracy, these single-modality approaches remain inherently limited. In particular, their reliance on EEG alone restricts the use of complementary physiological information and often leads to suboptimal performance in challenging sleep stages such as N1 and REM, where discriminative patterns are subtle and difficult to capture.

### 2.2. Multi-Modality Sleep Staging Methods

In parallel with the progress in single-modality approaches, multimodal electrophysiological sleep staging has evolved toward increasingly expressive architectures capable of leveraging the complementary characteristics of EEG, EOG, and EMG signals. Early efforts such as SleepPrintNet achieved an overall accuracy of approximately 88.8% on the MASS-SS3 dataset by jointly modeling multi-domain EEG features alongside EOG and EMG signals [21]. Subsequent works have explored more advanced fusion strategies. For instance, MMASleepNet reported accuracies of around 87.5% on Sleep-EDF [22], while MMS-SleepNet further improved performance, achieving approximately 89.8% on Sleep-EDF and 90.2% on SHHS [23]. Despite these improvements in overall performance, many existing multimodal approaches still rely on relatively coarse fusion strategies, such as feature concatenation or late-stage aggregation. These designs often process each modality independently before fusion, which limits their ability to explicitly capture fine-grained inter-modality dependencies. As a result, the complementary relationships between EEG and EOG are not fully exploited, particularly in sleep stages characterized by subtle cross-modal dynamics (e.g., N1 and REM).

To address these limitations, MFSleepNet introduces an interaction-based fusion strategy to model inter-modality dependencies, together with a gated temporal-channel attention mechanism to enhance informative features.

## 3. Materials and Methods

### 3.1. Dataset

In this study, a random subset of 55 subjects was selected from each dataset at the subject level for model development and evaluation. No demographic or clinical filtering was applied during subject selection.

#### 3.1.1. Sleep-EDF

The Sleep-EDF dataset is a publicly available collection of polysomnography (PSG) recordings contributed to PhysioNet by Bob Kemp [24,25]. Three channels were used as model inputs, including two EEG channels (Fpz–Cz and Pz–Oz) and one EOG channel. All signals were sampled at 100 Hz. Each sleep epoch was segmented into 30 s intervals, corresponding to 3000 sampling points per epoch.

#### 3.1.2. Sleep Heart Health Study

The Sleep Heart Health Study (SHHS) is a large-scale, multi-center cohort study initiated by the National Heart, Lung, and Blood Institute, aiming to investigate the cardiovascular and systemic effects of sleep-disordered breathing [26,27]. Four channels were adopted as model inputs, comprising two EEG channels (C3–A2 and C4–A1) and two EOG channels (EOG-L and EOG-R). EEG signals were sampled at 125 Hz, while EOG signals were sampled at 50 Hz. Following standard practice, all recordings were segmented into 30 s epochs for sleep staging.

#### 3.1.3. Human Sleep Project

The Human Sleep Project (HSP) dataset is a large-scale and continuously expanding collection of clinical PSG recordings [28]. Eight channels were used as model inputs, including six EEG channels (F3–M2, F4–M1, C3–M2, C4–M1, O1–M2, and O2–M2) and two EOG channels (E1–M2 and E2–M2). All signals were sampled or uniformly resampled to 200 Hz and measured in microvolts (µV). Similar to the other datasets, recordings were segmented into 30 s epochs for model training and evaluation.

### 3.2. Data Preprocessing

Prior to model training, all physiological signals underwent a standardized preprocessing pipeline to reduce inter-subject variability and mitigate low-frequency baseline drift. Considering the substantial amplitude and baseline differences across individuals, a linear detrending operation was applied to each channel by fitting and removing a first-order polynomial using a least-squares approach. This step removes slow baseline drifts introduced by electrode impedance fluctuations, perspiration, or gradual changes in recording conditions, while preserving physiologically meaningful oscillatory components [29]. Following detrending, all signals were normalized using z-score normalization on a per-recording basis, where the mean and standard deviation were computed independently for each recording [30]. No additional filtering or explicit signal cleaning procedures were applied, allowing the model to learn directly from minimally processed signals and better capture real-world data variability.

Notably, segments corresponding to body movements or other abnormal physiological activities were not explicitly excluded during preprocessing, enabling the model to be trained on more realistic and heterogeneous data. Through this preprocessing pipeline, inter-subject differences in baseline level and amplitude are effectively controlled, ensuring that the downstream model focuses on information relevant to sleep staging.

### 3.3. Model

#### 3.3.1. Overview of the Model

**Figure 1:**
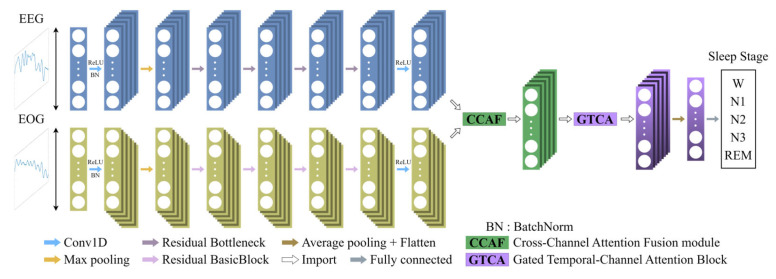
Overall architecture of the proposed MFSleepNet.

In this study, a Multimodal Fusion Network (MFSleepNet) is proposed for EEG-EOG-based sleep staging. The framework consists of three major components: (1) a Feature Extraction Backbone, (2) a Cross-Channel Attention Fusion Module, and (3) a gated temporal-channel attention block. Raw EEG and EOG segments are first processed by two modality-specific residual networks, which serve as the Feature Extraction Backbone to learn discriminative intra-modality representations from each modality. Since each dataset provides different numbers of EEG and EOG channels, this backbone operates directly on available modality-specific inputs without requiring channel alignment, thereby naturally accommodating heterogeneous channel configurations across datasets. The resulting EEG and EOG feature sequences are subsequently integrated through the Cross-Channel Attention Fusion Module to achieve multimodal information interaction. The fused representation is further refined by the gated temporal-channel attention block, which selectively emphasizes salient temporal dependencies and informative channel responses. The refined features are then fed into a classification head to generate sleep stage predictions. The selection of backbone networks and key hyperparameters is based on prior studies and empirical evaluation [3]. The overall architecture of MFSleepNet is illustrated in Figure 1.

#### 3.3.2. Feature Extraction Backbone

The Feature Extraction Backbone is designed to learn discriminative intra-modality representations from raw EEG and EOG signals before multimodal fusion. Formally, the inputs are denoted as $X_{EEG}\in R^{Ceeg\times L}$, $X_{EOG}\in R^{Ceog\times L}$, where *C* represents the number of channels and L denotes the temporal length. Each input is first processed by an identical initial stem composed of a 1D convolution (Conv), batch normalization (BN), a ReLU activation function, and a max-pooling layer, expressed as:

(1) $$X^{\prime }=MaxPool(\sigma (BN(Conv_{7\times 1}(X)))),$$

where σ(⋅) denote the ReLU functions.

**Figure 2:**
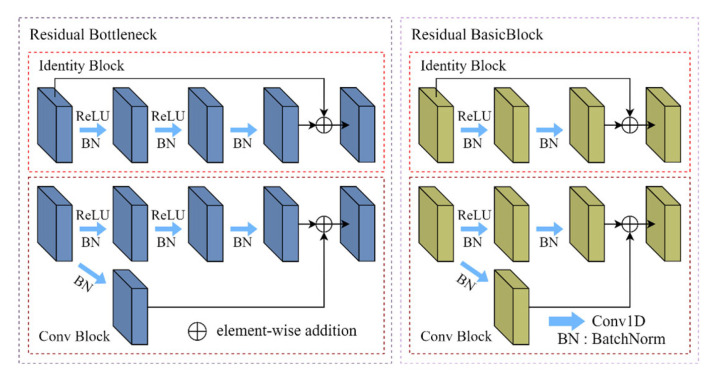
Residual structures of the EEG and EOG branches.

After the initial projection, the EEG and EOG feature maps are fed into modality-specific residual networks. Owing to the differences in temporal dynamics and spectral complexity between EEG and EOG signals, two customized residual architectures are employed [31]. For EEG, a Bottleneck-based residual structure (Figure 2) is adopted to provide a larger effective receptive field and deeper feature abstraction capability. The residual mapping of the Bottleneck block is expressed as:

(2) $$F_{bottleneck}(X)=Conv_{1\times 1}(X)\to Conv_{3\times 1}(X)\to Conv_{1\times 1}(X).$$

For EOG, which exhibits lower spectral complexity and smoother temporal variations, a lighter BasicBlock configuration is used (Figure 2). Its residual mapping is defined as:

(3) $$F_{basic}(X)=Conv_{3\times 1}(X)\to Conv_{3\times 1}(X).$$

Both modality-specific backbones consist of four stages with progressively increased feature dimensions and reduced temporal resolutions. Each stage begins with a stride-2 convolution when downsampling is required. Despite architectural differences, both networks output features at a temporal resolution of *L*/32.

At the end of both backbones, a shared final 1 × 1 convolution is applied to project the outputs of the EEG and EOG branches into a unified embedding dimension, yielding modality-aligned representations that serve as inputs to the subsequent fusion module. The process can be calculated by (4).

(4) $$f=Conv_{1\times 1}(X_{stage4}).$$

The detailed layer configuration of the Feature Extraction Backbone, including convolution parameters, residual block counts, and output dimensions for both EEG and EOG branches, is summarized in Table 1.

#### 3.3.3. Multimodal Feature Fusion Module

**Figure 3:**
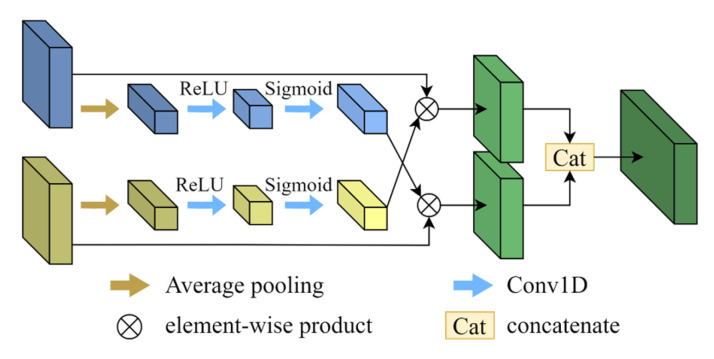
Architecture of the multimodal feature fusion module.

To exploit the complementary characteristics of EEG and EOG signals, a multimodal fusion module is introduced to perform bidirectional cross-channel interaction between modality-specific representations. Unlike standard cross-attention mechanisms based on query–key–value formulations, the proposed method operates at the channel level without explicit similarity computation. Specifically, each modality generates channel-wise gating weights from the global descriptor of the counterpart modality, enabling mutual modulation. This design allows dynamic cross-modal interaction beyond static fusion methods such as concatenation, facilitating more effective integration of complementary information. The architectural diagram and detailed layer configuration of the module are presented in Figure 3 and Table 2, respectively.

As illustrated in Figure 3, the EEG and EOG feature maps, denoted as *f_EEG_* and *f_EOG_*, are first processed by an adaptive average pooling operation to extract global channel descriptors from each modality:

(5) $$f_{EEG}\;=AvgPool(f_{EEG}\;),\;f_{EOG}\;=AvgPool(f_{EOG}).$$

These descriptors are then passed through two lightweight channel-transformation pathways, each consisting of a pair of 1 × 1 convolutions with a channel-reduction ratio, followed by ReLU and Sigmoid activations, to produce the cross-modal attention weights:

(6) $$\left\{\begin{array}{l} w_{EOG\to EEG}\;=\delta (Conv_{1\times 1}(\sigma (Conv_{1\times 1}(f_{EOG}))))\\ w_{EEG\to EOG}\;=\delta (Conv_{1\times 1}(\sigma (Conv_{1\times 1}(f_{EEG})))) \end{array}\right.,$$

where σ(⋅) and δ(⋅) denote the ReLU and Sigmoid functions, respectively.

The resulting weights are subsequently applied to the temporal feature maps of the opposite modality to obtain cross-modulated representations:

(7) $${\bar{f}}_{EEG}\;=f_{EEG}⊗w_{EOG\to EEG},\;{\bar{f}}_{EOG}=f_{EOG}⊗w_{EEG\to EOG},$$

where ⊗ represents element-wise multiplication.

Finally, the modulated features are concatenated along the channel dimension to form the fused representation:

(8) $$f_{fused}=Concat({\bar{f}}_{EEG},{\bar{f}}_{EOG}).$$

This fusion mechanism effectively captures inter-modal interactions by emphasizing informative channels and suppressing redundant modality-specific components, thereby providing a compact and semantically enriched representation for downstream feature refinement.

#### 3.3.4. Gated Temporal-Channel Attention Block

**Figure 4:**
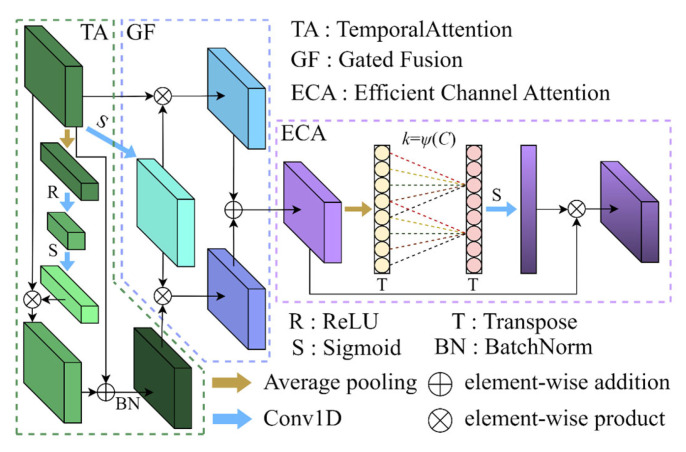
Architecture of the gated temporal-channel attention block.

To further mine channel-wise and temporal patterns contained in the fused representation, a gated temporal-channel attention block is applied to the fused feature map *f_fused_*. The block comprises three sequential components: (1) a Temporal Attention module that models dependencies along the time axis, (2) a gating-based fusion that adaptively merges original and temporally enhanced features, and (3) an Efficient Channel Attention (ECA) module that reinforces informative channels. The architectural diagram and detailed layer configuration are provided in Figure 4 and Table 3, respectively.

Specifically, the Temporal Attention submodule first aggregates temporal statistics via adaptive average pooling and computes channel-wise temporal weights through a lightweight bottleneck transform:

(9) $$z=AvgPool_{time}\left(f_{fused}\right),$$

(10) $$a=\delta (Conv_{1\times 1}(\sigma (Conv_{1\times 1}(z)))).$$

The temporally modulated feature is then obtained by channel-wise multiplication and a residual normalization:

(11) $$f_{temp}=BN(f_{fused}⊗a+f_{fused}).$$

A gating mechanism is subsequently used to adaptively fuse the original fused feature and the temporally enhanced feature. The gate is computed by a per-channel sigmoid activation of a 1 × 1 convolution applied to the original input:

(12) $$g=\sigma (Conv_{1\times 1}(f_{fused})),$$

and the gated fusion is formed as:

(13) $$f_{gate}=g⊗f_{fused}+\left(1-g\right)⊗f_{temp}.$$

Finally, an Efficient Channel Attention (ECA) module [32] is applied to *f_gate_* to capture local cross-channel interactions without heavy parameterization. The ECA operation performs global average pooling along the temporal axis to obtain channel descriptors, applies a lightweight 1D convolution with an adaptively determined kernel size k, and rescales the channels via a sigmoid activation:

(14) $$F=f_{gate}⊗\sigma (Conv_{1\times k}(AvgPool_{time}(f_{gate}))),$$

yielding the refined output F. The proposed block thus performs complementary temporal enhancement and lightweight channel recalibration while adaptively balancing original and enhanced information through gating.

#### 3.3.5. Classification Head

Following the refinement process in the gated temporal-channel attention block, the enhanced feature map F is passed through a global average pooling layer to aggregate temporal information into a compact representation. The pooled features are then flattened into a vector and fed into a fully connected layer for classification. A softmax activation function is applied to generate class probabilities. The detailed layer configuration is summarized in Table 4.

To supervise model training, the standard cross-entropy loss is employed.

### 3.4. Implementation Details

In this study, data from 5 randomly selected subjects were reserved for external validation, while data from the remaining 50 subjects were used for model performance evaluation. For the 50-subject dataset, all epochs were pooled together and a stratified 5-fold cross-validation strategy was applied at the epoch level. Specifically, the combined dataset was randomly partitioned into five equal-sized subsets, without enforcing subject-wise separation. In each iteration, four subsets were used for training and one subset was used for testing. The final reported results represent the average performance across all five folds.

During training, the order of samples in the training set was randomly shuffled to enhance generalization. The model was optimized using the Adam optimizer with an initial learning rate of 1 × 10^−4^. To mitigate overfitting, L2 regularization was applied via a weight decay of 1 × 10^−4^. The model was trained for 100 epochs with a batch size of 32. Validation was performed at the end of each epoch, and the model checkpoint with the best validation accuracy was retained for final testing. Hyperparameters were determined via a limited grid search, where the learning rate was selected from {1 × 10^−3^, 1 × 10^−4^, 1 × 10^−5^} and the weight decay from {1 × 10^−5^, 1 × 10^−4^, 1 × 10^−3^}.

The experimental platform for this study is based on the Windows 10 operating system, equipped with a GeForce RTX 4090 D GPU (24 GB VRAM), an Intel^®^ Core (TM) i9-14900K CPU @ 3.20 GHz, and 32 GB of RAM. The development environment for signal fusion and feature fusion used Matlab 2020b. The development environment for model building, training and testing used Pycharm 2024.1.7; the DL framework used PyTorch 2.4.0; the parallel computing framework used CUDA version 12.4; the Python version used is 3.9.

### 3.5. Evaluation Metrics

In this study, overall accuracy (OA), Cohen’s Kappa coefficient (κ) and Macro-F1 (MF1) were adopted to evaluate the performance of the proposed model. Specifically, OA represents the proportion of correctly predicted samples relative to the total number of samples. κ is widely used in medical diagnostics as a statistical measure to assess the agreement between the automatic sleep staging results generated by the model and those classified by sleep experts [33]. MF1 is introduced to provide a balanced evaluation across classes by assigning equal importance to each sleep stage, making it more suitable for imbalanced datasets. Given the true positives (TP), false positives (FP), true negatives (TN), and false negatives (FN) for the c-th class, OA, κ and MF1 are formally defined as follows:

(15) $$\mathrm{OA}=\sum_{c=1}^{5}\frac{TP_{c}+TN_{c}}{TP_{c}+TN_{c}+FP_{c}+FN_{c}}\times 100\%,$$

(16) $$\kappa =\frac{P_{0}-P_{e}}{1-P_{e}},$$

(17) $$MF1=\frac{1}{C}\sum_{c=1}^{C}F1_{c},$$

where *N_c_* represents the number of samples for the c-th class; *P*_0_ represents the observed agreement, $P_{0}=\;\mathrm{OA}$, and *P*_e_ represents the expected agreement, $P_{e}=\;\frac{TP_{c}+FN_{c}}{TP_{c}+TN_{c}+FP_{c}+FN_{c}}\times \frac{TP_{c}+FP_{c}}{TP_{c}+TN_{c}+FP_{c}+FN_{c}}+\frac{TN_{c}+FP_{c}}{TP_{c}+TN_{c}+FP_{c}+FN_{c}}\times \frac{TN_{c}+FN_{c}}{TP_{c}+TN_{c}+FP_{c}+FN_{c}}$; *C* denotes the number of sleep stages. $F1_{c}=\;\frac{2\times Precision_{c}\times Recall_{c}}{Precision_{c}+Recall_{c}}$, $Precision_{c}=\;\frac{TP_{c}}{TP_{c}+FP_{c}}$, and $Recall_{c}=\;\frac{TP_{c}}{TP_{c}+FN_{c}}$.

## 4. Experiments and Results

### 4.1. Comparison with State-of-the-Art Methods

**Figure 5:**
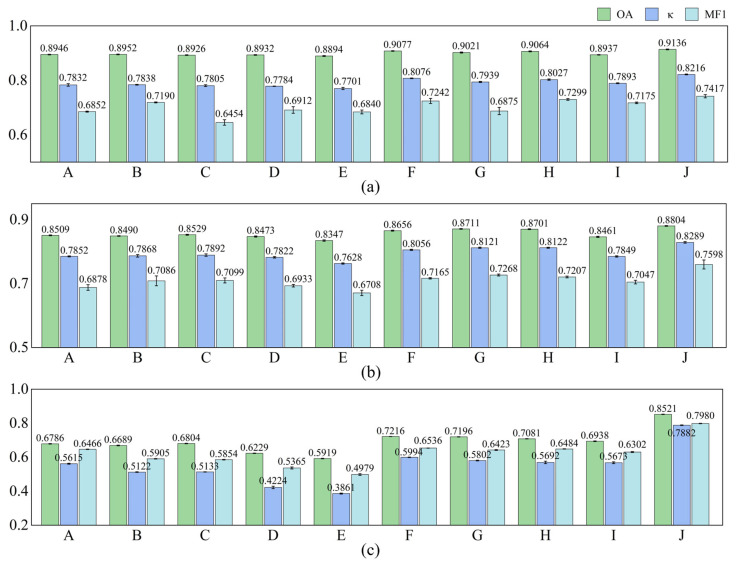
Performance comparison of different sleep staging methods on three datasets: (**a**) Sleep-EDF, (**b**) SHHS, and (**c**) HSP. Note: Labels A–J correspond to CSCNN, AttnSleep, SleepContextNet, MultiScaleSleepNet, SleepHybridNet, SleepPrintNet, MSDFN, MMASleepNet, MMS-SleepNet, and MFSleepNet, respectively. Error bars indicate the standard deviation across cross-validation folds.

In this section, the proposed MFSleepNet is compared with several representative and recently reported sleep staging methods on the datasets used in this study. The comparison includes five single-modality EEG-based models (CSCNN [16], AttnSleep [17], SleepContextNet [18], MultiScaleSleepNet [19], and SleepHybridNet [20]) and four multimodal models (SleepPrintNet [21], MSDFN [34], MMASleepNet [23], and MMS-SleepNet [24]). All baseline methods were re-implemented and evaluated under identical preprocessing, data partitioning, and input configurations to ensure a fair comparison. The experimental results are shown in Figure 5.

According to Figure 5, on the Sleep-EDF dataset, the single-modality models achieve overall accuracies ranging from 88.94% to 89.52%, with corresponding κ between 0.7701 and 0.7838, and MF1 ranging from 0.6454 to 0.7190. In contrast, existing multimodal methods generally exhibit higher performance, with OA values spanning 89.37% to 90.77%, κ values from 0.7893 to 0.8076, and MF1 from 0.6875 to 0.7299. Notably, the proposed MFSleepNet achieves the best performance on this dataset, obtaining an OA of 91.36%, a κ of 0.8216, and an MF1 of 0.7417, outperforming all compared single-modality and multimodal baselines.

A similar performance trend can be observed on the SHHS dataset. The single-modality models yield OA values in the range of 83.47% to 85.09%, while existing multimodal approaches achieve higher accuracies, reaching up to 87.11%, with corresponding κ and MF1 values also showing consistent improvements. In comparison, MFSleepNet further improves the performance, achieving the highest OA of 88.04%, κ of 0.8289, and MF1 of 0.7598 among all evaluated methods.

On the more challenging HSP dataset, the performance gap between single-modality and multimodal models becomes more pronounced. This may be attributed to the increased input dimensionality and more complex inter-channel relationships in HSP, which make feature extraction and multimodal fusion more challenging. The OA of single-modality methods ranges from 59.19% to 67.86%, whereas existing multimodal models achieve accuracies between 69.38% and 72.16%, with corresponding κ and MF1 values following similar trends. In this setting, MFSleepNet shows a substantial advantage, achieving the highest OA of 85.21%, κ of 0.7882, and MF1 of 0.7980.

Overall, across all three datasets, the proposed MFSleepNet consistently achieves the highest OA, κ, and MF1 among all compared methods, demonstrating its superior performance and strong generalization capability across diverse sleep staging benchmarks. The reported values represent the mean ± standard deviation across cross-validation folds.

### 4.2. Ablation Study

**Figure 6:**
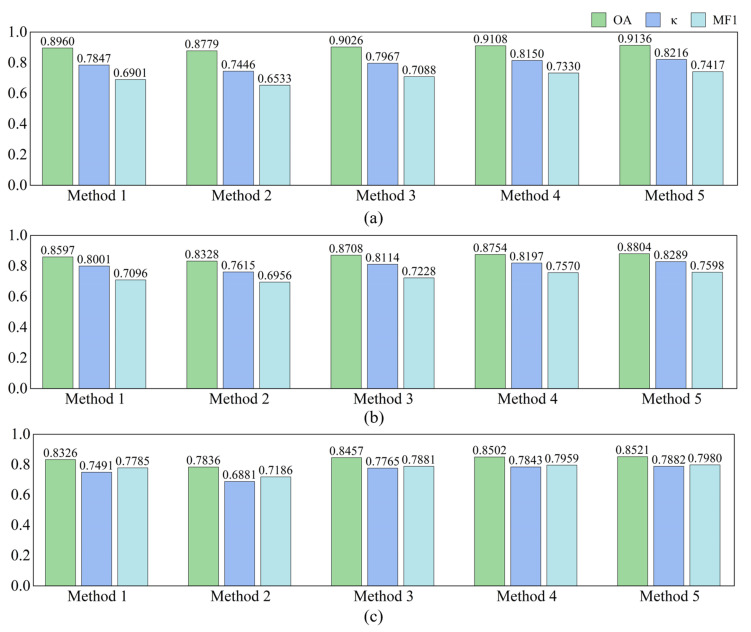
Ablation study results of different MFSleepNet variants on three datasets: (**a**) Sleep-EDF, (**b**) SHHS, and (**c**) HSP.

To comprehensively evaluate the effectiveness of each component in the proposed MFSleepNet, a series of ablation experiments were conducted. Five variants were designed: Method 1 utilizes only the EEG branch, Method 2 employs only the EOG branch, Method 3 combines EEG and EOG branches using direct feature concatenation, Method 4 further incorporates the multimodal feature fusion module on top of Method 3, and Method 5 additionally integrates the gated temporal-channel attention block, corresponding to the complete MFSleepNet architecture. All ablation experiments were conducted on the Sleep-EDF, SHHS, and HSP datasets. The quantitative results are illustrated in Figure 6.

According to Figure 6, a clear performance progression can be observed as additional modalities and fusion modules are introduced. On the Sleep-EDF dataset, Method 1 achieves an OA of 89.60% with a κ of 0.7847, while Method 2 yields lower performance, with an OA of 87.79% and a κ of 0.7446. When EEG and EOG features are jointly utilized in Method 3, the OA increases to 90.26% and κ to 0.7967. Further improvements are obtained by incorporating the multimodal feature fusion module in Method 4, achieving an OA of 91.08% and κ of 0.8150. The complete MFSleepNet (Method 5) attains the best performance, with an OA of 91.36% and κ of 0.8216.

A similar trend is observed on the SHHS dataset. The single-modality configurations (Method 1 and Method 2) achieve OA values of 85.97% and 83.28%, respectively, while Method 3 improves the OA to 87.08%. Method 4 and Method 5 further enhance performance, reaching OA values of 87.54% and 88.04%, with corresponding κ values increasing consistently from 0.8001 to 0.8289. On the HSP dataset, the performance gap between single-modality and multimodal methods becomes more evident. Method 1 and Method 2 obtain OA values of 83.26% and 78.36%, respectively, whereas Method 3 improves the OA to 84.57%. Additional gains are achieved by Method 4 (85.02%) and Method 5 (85.21%), accompanied by a steady increase in κ from 0.7491 to 0.7882.

**Figure 7:**
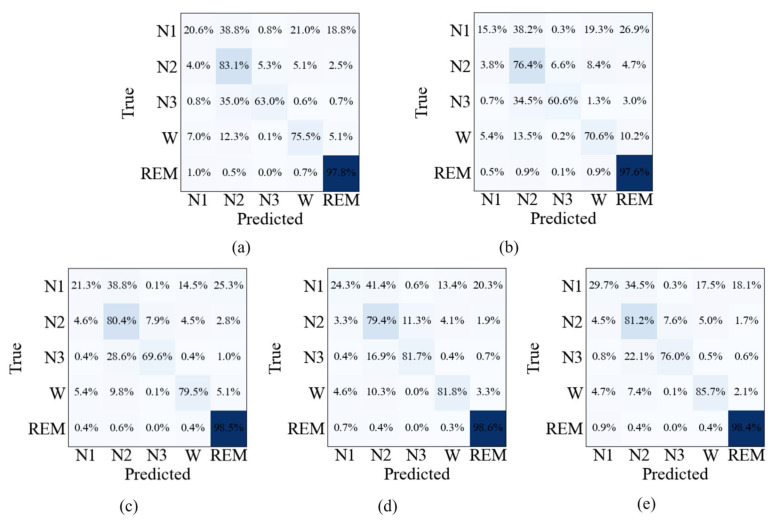
Confusion matrix of different ablation variants on the Sleep-EDF dataset: (**a**) Method 1, (**b**) Method 2, (**c**) Method 3, (**d**) Method 4, and (**e**) Method 5. Note: Darker colors indicate higher percentages.

**Figure 8:**
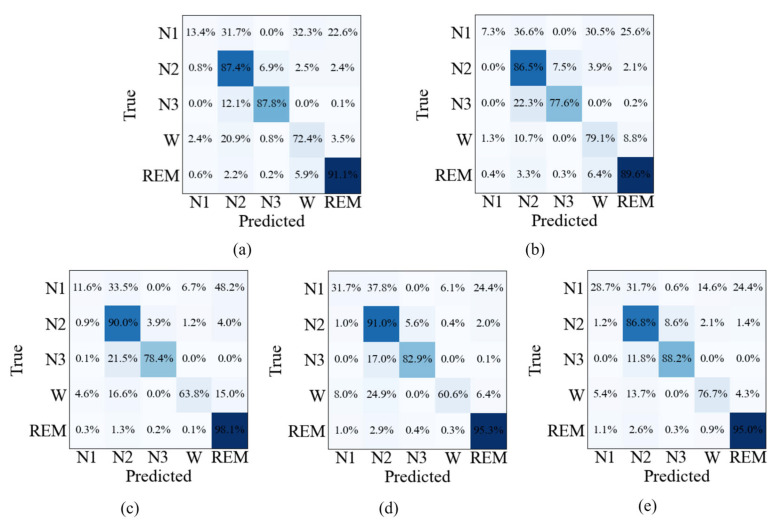
Confusion matrix of different ablation variants on the SHHS dataset: (**a**) Method 1, (**b**) Method 2, (**c**) Method 3, (**d**) Method 4, and (**e**) Method 5. Note: Darker colors indicate higher percentages.

**Figure 9:**
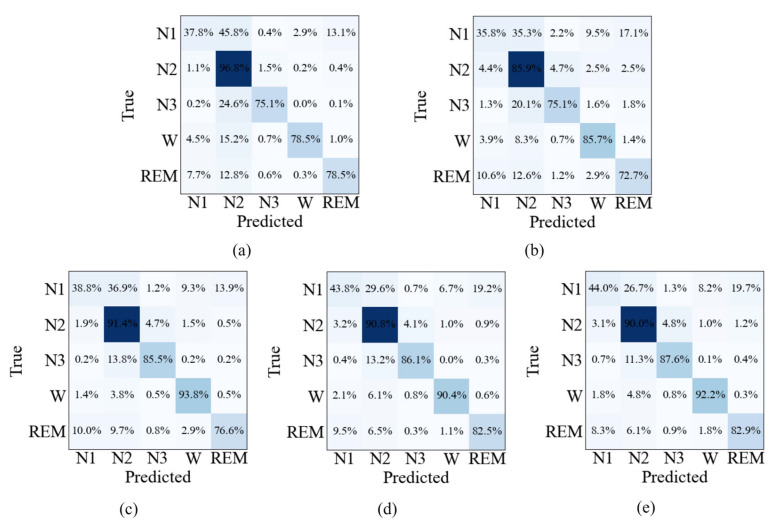
Confusion matrix of different ablation variants on the HSP dataset: (**a**) Method 1, (**b**) Method 2, (**c**) Method 3, (**d**) Method 4, and (**e**) Method 5. Note: Darker colors indicate higher percentages.

The confusion matrices shown in Figure 7, Figure 8 and Figure 9 provide further insight into the stage-wise classification behavior of different ablation settings. For the single-modality models, Method 1 exhibits relatively higher correct predictions in N2 and N3 stages, while Method 2 shows comparatively better performance in the Wake stage. When both modalities are combined in Method 3, improvements are observed across most sleep stages compared with either single-modality configuration. With the introduction of the multimodal feature fusion module in Method 4, further gains are achieved in per-class predictions for nearly all stages. The full model (Method 5) generally achieves the best performance across the majority of sleep stages on all three datasets. Notably, lower recognition performance is consistently observed in the N1 stage across all methods, reflecting the inherent difficulty of this stage.

### 4.3. EEG–EOG Correlation Analysis and Inter-Modality Interaction Effects

**Figure 10:**
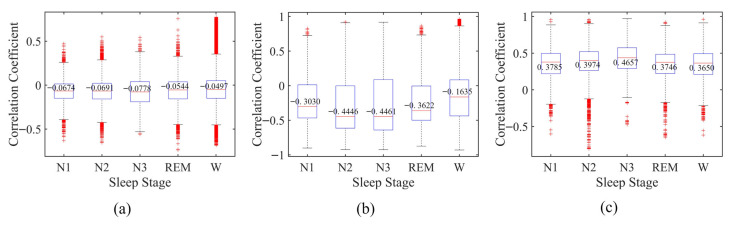
Spearman rank correlation analysis between EEG and EOG signals across different sleep stages on three datasets: (**a**) Sleep-EDF, (**b**) SHHS, and (**c**) HSP.

To further investigate EEG–EOG relationships across sleep stages, a Spearman rank correlation analysis [35] was conducted, as summarized in Figure 10. The absolute correlation value reflects the strength of monotonic association between the two modalities and was computed on a per-epoch basis using preprocessed 30-s EEG and EOG signals (Section 3.1).

On the Sleep-EDF dataset, the median correlation coefficients for N1, N2, N3, REM, and Wake are −0.0674, −0.0691, −0.0778, −0.0544, and −0.0497, respectively, with N3 showing the largest absolute value. Similar stage-dependent patterns are observed on SHHS and HSP, where deeper sleep stages (particularly N2 and N3) tend to exhibit relatively higher absolute correlation values. The Kruskal–Wallis test [36] indicates statistically significant differences in correlation distributions across sleep stages for all datasets (*p* < 10^−16^), consistent with previous observations on physiological variability [37].

To evaluate the effect of inter-modality interaction, the performance of EEG-only and EOG-only branches is further reported before and after feature interaction (Table 5). Across all datasets, both branches show consistent improvements after interaction. On Sleep-EDF, EEG improves from 89.60% to 90.03% OA, while EOG increases from 87.79% to 89.18% OA. Similar improvements are observed on SHHS and HSP in terms of OA and κ.

Overall, the ablation results demonstrate a consistent performance improvement across all datasets as additional modalities and modules are introduced. Although the magnitude of improvement is relatively modest in some cases, the overall trend remains stable across datasets and evaluation metrics.

Overall, these results provide empirical evidence that inter-modality interaction consistently benefits feature representation learning across different datasets and modalities.

### 4.4. Grad-CAM–Based Visualization and Interpretability Analysis

To provide an intuitive interpretation of the decision-making process of MFSleepNet, a Grad-CAM–based visualization analysis [38] was conducted. Specifically, feature activation maps were generated for three representative components: the EEG branch feature maps (Feature Map 1), the EOG branch feature maps (Feature Map 2), and the output feature maps of the gated temporal-channel attention block (Feature Map 3). The resulting heatmaps use a color gradient to indicate activation intensity, ranging from black (negligible activation) through red and yellow to white (highest activation), highlighting regions that contribute most strongly to the model’s predictions.

**Figure 11:**
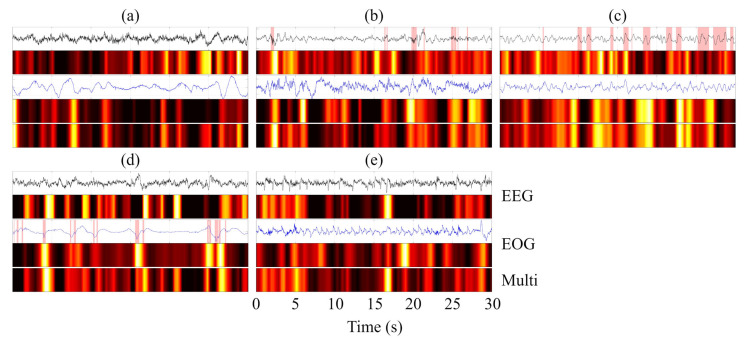
Grad-CAM visualization of EEG and EOG signals across five sleep stages (N1, N2, N3, REM, and W) on the Sleep-EDF dataset. Panels (**a**–**e**) correspond to N1, N2, N3, REM, and W, respectively.

**Figure 12:**
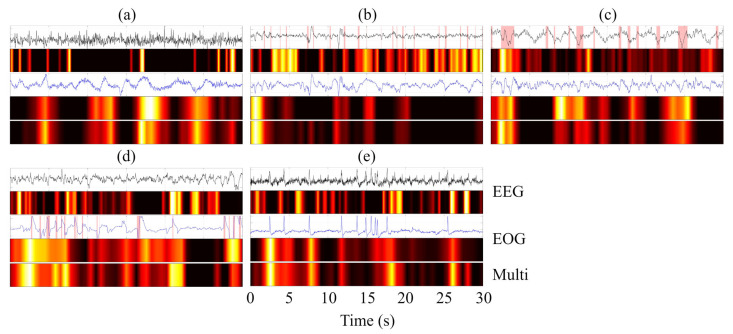
Grad-CAM visualization of EEG and EOG signals across five sleep stages (N1, N2, N3, REM, and W) on the SHHS dataset. Panels (**a**–**e**) correspond to N1, N2, N3, REM, and W, respectively.

**Figure 13:**
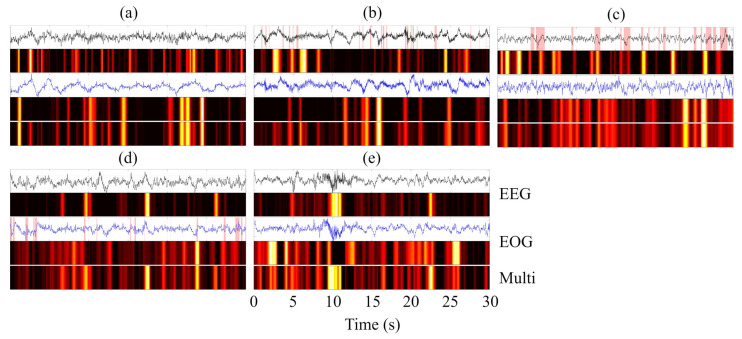
Grad-CAM visualization of EEG and EOG signals across five sleep stages (N1, N2, N3, REM, and W) on the HSP dataset. Panels (**a**–**e**) correspond to N1, N2, N3, REM, and W, respectively.

The visualization analysis was performed for all sleep stages across the three datasets. For each case, the Grad-CAM heatmaps were aligned with the corresponding raw EEG and EOG signals within a single 30 s epoch, facilitating direct temporal comparison, as illustrated in Figure 11, Figure 12 and Figure 13. In the waveform visualizations, the red shaded regions denote characteristic physiological event segments within each 30 s epoch, corresponding to sleep spindles in N2, slow-wave (δ) activity in N3, and burst-like eye movement events in REM.

Across datasets and sleep stages, the activation regions in Feature Maps 1 and 2 are generally well aligned with time segments exhibiting pronounced amplitude variations in the EEG and EOG signals, indicating that the model attends preferentially to temporally salient signal patterns rather than uniformly processing the entire epoch. Beyond general amplitude sensitivity, stage-specific physiological characteristics are also reflected in the activation patterns [39,40,41]. For instance, in the N2 stage, high-activation regions in the EEG branch often coincide with spindle-like oscillatory activity, while during N3, the EEG-related activations are predominantly concentrated in segments dominated by slow-wave (δ) activity. In the REM stage, the EOG branch exhibits strong activations aligned with burst-like eye movement events, suggesting sensitivity to characteristic ocular dynamics.

Compared with the modality-specific feature maps, Feature Map 3 exhibits a more compact and selective activation pattern. This indicates that the fused representation emphasizes a subset of discriminative temporal regions jointly informed by EEG and EOG features, effectively filtering out less relevant responses while preserving task-relevant information. Overall, the Grad-CAM visualizations illustrate a clear progression from modality-specific saliency detection to refined and integrated feature representations, providing qualitative evidence that the proposed architecture captures physiologically meaningful patterns and consolidates them into a focused representation for sleep stage classification.

### 4.5. Cross-Subject Evaluation and Subject-Adaptive External Validation

**Figure 14:**
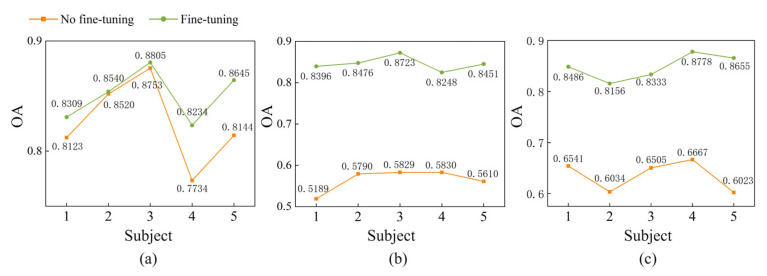
External validation results of MFSleepNet under subject-independent settings on three datasets: (**a**) Sleep-EDF, (**b**) SHHS, and (**c**) HSP.

To evaluate the performance of the proposed MFSleepNet on unseen subjects, an external validation experiment was conducted as described in the experimental setup. The corresponding results are illustrated in Figure 14.

First, the trained model was directly applied to the held-out subjects without any subject-specific adaptation. On the Sleep-EDF dataset, the OA obtained for the five subjects is 81.23%, 85.20%, 87.53%, 77.34%, and 81.44%, respectively. On the SHHS dataset, the corresponding OA values are 51.89%, 57.90%, 58.29%, 58.30%, and 56.10%. For the HSP dataset, the OA values achieved are 65.41%, 60.34%, 65.05%, 66.67%, and 60.23%, respectively. These results indicate noticeable performance variability and degradation when the model is directly transferred to unseen individuals, reflecting the challenges posed by inter-subject variability.

To further address this issue, a lightweight fine-tuning strategy was adopted [42,43,44]. For each unseen subject, the available data were stratified and randomly split with a ratio of 1:9, where 10% of the data were used for model adaptation and the remaining 90% for testing. During fine-tuning, only the parameters of the classification head were updated, while all feature extraction modules were kept fixed, allowing subject-specific decision boundaries to be adjusted while preserving the learned shared representations.

After fine-tuning, consistent performance improvements are observed across all three datasets. On the Sleep-EDF dataset, the OA values for the five subjects increase to 83.09%, 85.40%, 88.05%, 82.34%, and 86.45%, respectively. For the SHHS dataset, the corresponding OA values reach 83.96%, 84.76%, 87.23%, 82.48%, and 84.51%. On the HSP dataset, the OA values further improve to 84.86%, 81.56%, 83.33%, 87.78%, and 86.55%, respectively.

These results indicate that the model exhibits a certain level of baseline generalization to unseen subjects, while performance is influenced by inter-subject variability. With limited subject-specific adaptation, consistent improvements can be achieved across different individuals.

## 5. Discussion

In this study, a multimodal sleep staging framework, termed MFSleepNet, was developed to address challenges in physiological signal fusion, interpretability, and generalization to unseen subjects. By jointly integrating EEG and EOG signals through an interactive fusion strategy and gated temporal-channel signal weighting, the proposed framework exploits complementary electrophysiological information while adaptively emphasizing informative temporal segments and signal channels relevant to sleep dynamics.

To evaluate measurement performance and robustness, Section 4.1 compares MFSleepNet with representative sleep staging methods. Experimental results on three public datasets demonstrate that MFSleepNet consistently achieves the highest OA, κ, and MF1 among all compared approaches. These improvements can be attributed to effective multimodal integration and the structured design of the proposed fusion and weighting mechanisms. Compared with EEG-only methods, incorporating EOG provides complementary information that improves discrimination between sleep stages, particularly those with similar EEG patterns. Furthermore, the interaction-based fusion module enables explicit modeling of cross-modality dependencies, while the temporal-channel weighting mechanism highlights informative signal segments and channel relationships. Together, these components contribute to improved robustness across datasets with varying characteristics. It is also observed that MF1 scores are generally lower than OA and κ across all methods. This is mainly due to the inherent class imbalance in sleep staging datasets, where certain stages are underrepresented and more difficult to predict. Despite this challenge, MFSleepNet still achieves consistent improvements in MF1, indicating its effectiveness in handling imbalanced data and capturing complementary cross-modal information.

The ablation results demonstrate consistent improvements across all three datasets as different modules are progressively introduced, indicating that the proposed architecture is stable and robust under varying conditions. The comparison between single-modality and multimodal configurations shows that EEG and EOG provide complementary physiological information, and their joint modeling yields more informative representations than either modality alone, highlighting the benefit of cross-modal dependency modeling. The interaction-based fusion module further improves performance by explicitly capturing inter-modality interactions, while the gated temporal-channel attention mechanism consistently enhances results by emphasizing informative temporal and channel-wise features. These improvements are reflected not only in overall performance but also in minority-class representation. In particular, for the N1 stage, the proposed modules yield consistent gains in recall compared with single-modality and simple fusion baselines across all datasets. Although the absolute performance on N1 remains modest, the observed improvements indicate that cross-modal interaction and adaptive feature refinement contribute to enhanced modeling of minority-class patterns. In addition, the relatively strong performance of the EOG-only configuration can be attributed to discriminative eye-movement patterns, which have been shown in prior studies (e.g., SleepPrintNet [21]) to exhibit clear stage-dependent regularity, especially in REM sleep. Overall, these findings indicate that each component contributes incrementally to performance improvement, supporting the effectiveness and rationality of the proposed framework.

Beyond performance metrics, Section 4.3 provides correlation-based and interaction-based analyses of multimodal fusion. The Spearman results indicate stage-dependent variations in EEG–EOG associations, with statistically significant inter-stage differences (*p* < 10^−16^). However, the correlation magnitudes differ across datasets: they are relatively weak in Sleep-EDF (≈0.07) but moderate in SHHS and HSP (≈0.4). This discrepancy may be related to differences in sample size, channel configuration, and underlying physiological variability. Despite these differences, the observed correlations should be interpreted with caution, as they may also be influenced by shared noise, recording conditions, or preprocessing factors, rather than reflecting strong physiological coupling. Importantly, these correlations are not intended to explain the observed performance improvements. Instead, they serve as a descriptive analysis of potential cross-modal variability. The effectiveness of the proposed method is primarily attributed to the interaction-based fusion mechanism and the gated temporal-channel attention module, which enable flexible integration of complementary information in a data-driven manner. Consistent with this design, the interaction-based experiments demonstrate that explicitly modeling inter-modality dependencies improves the performance of both EEG-only and EOG-only branches after fusion. This suggests that, even in the presence of weak or moderate statistical associations, cross-modality interactions can still provide useful complementary information for sleep stage classification.

Grad-CAM–based visualization further provides qualitative insight into the measurement process. The highlighted regions indicate that the framework selectively focuses on physiologically meaningful and temporally salient signal segments, rather than uniformly processing entire epochs. The transition from modality-specific activations to more compact and selective responses after temporal-channel weighting suggests effective consolidation of EEG and EOG information. This behavior improves interpretability and offers an intuitive explanation for the observed performance gains, particularly in distinguishing sleep stages with subtle and overlapping electrophysiological patterns.

External validation on unseen subjects provides additional insights into cross-subject performance. When directly applied to new individuals, a noticeable performance degradation is observed, reflecting substantial inter-subject variability in physiological signals. This also indicates that the current setting does not represent strict subject-independent generalization. Under this condition, only limited baseline generalization is observed. By further applying a lightweight subject-specific calibration strategy—where only the classification head is updated while the feature extraction backbone is fixed—consistent performance improvements are achieved across all datasets. This suggests that the learned representations retain partial transferability, while inter-subject differences are mainly manifested as distribution shifts that can be mitigated through decision boundary adaptation. Overall, these results highlight the trade-off between cross-subject generalization and subject-specific adaptation.

Despite these encouraging results, several limitations remain. First, the use of an epoch-level cross-validation strategy during internal evaluation may introduce subject-level information mixing. Although external validation on unseen subjects is conducted, strict subject-independent generalization remains challenging due to substantial inter-subject variability. Second, while the proposed lightweight calibration strategy improves subject adaptation, it relies on a small amount of labeled subject-specific data. Developing more label-efficient or fully unsupervised adaptation strategies remains an important direction for future work. In addition, the inherent class imbalance in sleep staging datasets may lead to performance disparities across different classes, particularly affecting minority stages. Future work will focus on improving cross-subject generalization, reducing dependence on labeled subject-specific data, and enhancing robustness across all sleep stages.

## 6. Conclusions

In this study, a multimodal sleep staging framework, termed MFSleepNet, was developed to address challenges in physiological signal fusion, interpretability, and cross-subject variability. By jointly integrating EEG and EOG signals, the proposed framework combines interactive multimodal feature fusion with gated temporal-channel signal weighting, enabling explicit modeling of inter-modality dependencies and adaptive emphasis on informative temporal segments and signal channels. Experimental results on three public datasets under an epoch-level evaluation protocol demonstrate that MFSleepNet consistently outperforms representative single-modality and multimodal baseline methods in terms of overall accuracy and inter-rater agreement, while ablation studies verify the contribution of each functional module. Correlation analysis and interaction-based experiments indicate stage-dependent EEG–EOG variations, providing complementary insights into cross-modal relationships. In addition, Grad-CAM visualizations show alignment between highlighted signal regions and physiologically meaningful patterns, enhancing interpretability. External evaluation on unseen subjects reveals a noticeable performance drop, highlighting limited baseline generalization due to inter-subject variability. A lightweight subject-specific adaptation strategy, which updates only the classification head, is shown to improve performance and operates with a small amount of labeled subject-specific data.

Overall, MFSleepNet provides an effective and interpretable solution for multimodal sleep staging. Future work will focus on addressing these limitations by exploring stricter subject-independent evaluation protocols, developing more label-efficient or unsupervised adaptation strategies to reduce reliance on subject-specific annotations, and improving robustness under class imbalance and inter-subject variability in practical sleep staging scenarios.

## Figures and Tables

**Table 1:** Layer configuration of the feature extraction backbone.

	Layer Type			Parameters	Output Shape	Signal
Feature Extraction Backbone	Stem	Input		-	(*B*, *C*, *L*)	EEG
		Conv1D		*k* = 7, *s* = 2, *p* = 3	(*B*, 64, *L*/2)	
		Max Pool		*k* = 3, *s* = 2, *p* = 1	(*B*, 64, *L*/4)	
	Stage 1	Residual Bottleneck	×3	*k* = 1, *s* = 1	(*B*, 256, *L*/4)	
				*k* = 3, *s* = 1, *p* = 1		
				*k* = 1, *s* = 1		
	Stage 2	Residual Bottleneck	×4	*k* = 1, *s* = 1	(*B*, 512, *L*/8)	
				*k* = 3, *s* = 2/1, *p* = 1		
				*k* = 1, *s* = 1		
	Stage 3	Residual Bottleneck	×6	*k* = 1, *s* = 1	(*B*, 1024, *L*/16)	
				*k* = 3, *s* = 2/1, *p* = 1		
				*k* = 1, *s* = 1		
	Stage 4	Residual Bottleneck	×3	*k* = 1, *s* = 1	(*B*, 2048, *L*/32)	
				*k* = 3, *s* = 2/1, *p* = 1		
				*k* = 1, *s* = 1		
		Conv1D		*k* = 1, *s* = 1	(*B*, 1024, *L*/32)	
	Stem	Input		-	(*B*, *C*, *L*)	EOG
		Conv1D		*k* = 7, *s* = 2, *p* = 3	(*B*, 64, *L*/2) (*B*, 64, *L*/4)	
		Max Pool		*k* = 3, *s* = 2, *p* = 1		
	Stage 1	Residual BasicBlock	×2	*k* = 3, *s* = 1, *p* = 1	(*B*, 64, *L*/4)	
				*k* = 3, *s* = 1, *p* = 1		
	Stage 2	Residual BasicBlock	×2	*k* = 3, *s* = 2/1, *p* = 1 *k* = 3, *s* = 1, *p* = 1	(*B*, 128, *L*/8)	
	Stage 3	Residual BasicBlock	×2	*k* = 3, *s* = 2/1, *p* = 1	(*B*, 256, *L*/16)	
				*k* = 3, *s* = 1, *p* = 1		
	Stage 4	Residual BasicBlock	×2	*k* = 3, *s* = 2/1, *p* = 1	(*B*, 512, *L*/32)	
				*k* = 3, *s* = 1, *p* = 1		
		Conv1D		*k* = 1, *s* = 1	(*B*, 1024, *L*/32)	

**Table 2:** Layer configuration of the multimodal feature fusion module.

	Layer Type	Parameters	Output Shape	Signal
Multimodal feature fusion module	Input	-	(*B*, 1024, *L*/32)	EEG
	AdaptiveAvgPool1D	-	(*B*, 1024, 1)	
	Conv1D	*k* = 1	(*B*, 1024, 1)	
	Conv1D	*k* = 1	(*B*, 1024, 1)	
	Element-wise product	-	(*B*, 1024, *L*/32)	EOG
	Input	-	(*B*, 1024, *L*/32)	EOG
	AdaptiveAvgPool1D	-	(*B*, 1024, 1)	
	Conv1D	*k* = 1	(*B*, 1024, 1)	
	Conv1D	*k* = 1	(*B*, 1024, 1)	
	Element-wise product	-	(*B*, 1024, *L*/32)	EEG
	Concat	-	(*B*, 2048, *L*/32)	EEG-EOG

**Table 3:** Layer configuration of the gated temporal-channel attention block.

		Layer Type	Parameters	Output Shape
Gated temporal-channel attention block		Input	-	(*B*, 2048, *L*/32)
	Temporal Attention	AdaptiveAvgPool1D	-	(*B*, 2048, 1)
		Conv1D	*k* = 1	(*B*, 256, 1)
		Conv1D	*k* = 1	(*B*, 2048, 1)
		Element-wise product	-	(*B*, 2048, *L*/32)
	Gated Fusion	Conv1D	*k* = 1	(*B*, 2048, *L*/32)
		Element-wise product	-	(*B*, 2048, *L*/32)
		Element-wise addition	-	(*B*, 2048, *L*/32)
	Efficient Channel Attention	AdaptiveAvgPool1D	-	(*B*, 2048, 1)
		Transpose	-	(*B*, 1, 2048)
		Conv1d	*k* = 7, *s* = 1, *p* = 3	(*B*, 1, 2048)
		Transpose	-	(*B*, 2048, 1)
		Element-wise addition	-	(*B*, 2048, *L*/32)

**Table 4:** Layer configuration of the classification head.

	Layer Type	Parameters	Output Shape
Classification head	Input	-	(*B*, 2048, *L*/32)
	AdaptiveAvgPool1D	-	(*B*, 2048, 1)
	Flatten	-	(*B*, 2048)
	Linear	-	(*B*, 5)

**Table 5:** Performance comparison of EEG-only and EOG-only branches before and after inter-modality feature interaction on three datasets.

Dataset	Signal	OA (%)	
		Before Interaction	After Interaction
Sleep_edf	EEG	89.60	90.03
	EOG	87.79	89.18
SHHS	EEG	85.97	86.52
	EOG	83.28	85.14
HSP	EEG	83.26	83.93
	EOG	78.36	82.18

## Data Availability

Data will be made available on request.
